# Supplementary material for: Disease Occurrence and Climatic Factors Jointly Structure Pomelo Leaf Fungal Succession in Disturbed Agricultural Ecosystem
Source: Microorganisms. 2024 Jun 6;12(6):1157. doi: 10.3390/microorganisms12061157 (PMC11205469; doi:10.3390/microorganisms12061157)
Supplement: Supplementary file 1 [file microorganisms-12-01157-s001.zip › microorganisms-2948714-supplementary.pdf]

Table S1. The usage of fungicides in Chengjiang and Xiyang orchards.

Month	Orchard	Fungicide	Dilution fold
February 2021	Chengjiang	46% Copper hydroxide	750
		70% Sulfur·Thiram·Thiophanate-methyl	750
	Xiyang	32.5% Difenoconazole·Azoxystrobin	2500
		80% Mancozeb	600
March 2021	Chengjiang	70% Sulfur·Thiram·Thiophanate-methyl	750
		50% Tebuconazole	750
		25% Trifloxystrobin	750
	Xiyang	80% Mancozeb	600
		56% Mancozeb, 8% Oxadixyl	600
April 2021	Chengjiang	50% Tebuconazole, 25% Trifloxystrobin	750
		86% Bordeaux mixture	750
	Xiyang	46% Copper hydroxide	1500
		30% Difenoconazole·Propiconazol	3000
		46% Copper hydroxide	1500
		30% Difenoconazole·Propiconazol	3000
May 2021	Chengjiang	50% Sulfur·Carbendazim	750
		68.75% Famoxadone·Mancozeb	750
	Xiyang	20% Pydiflumetofen	2000
June 2021	Chengjiang	46% Copper hydroxide	750
		43% Mancozeb	750
July 2021	Chengjiang	50% Tebuconazole, 25% Trifloxystrobin	750
		80% Mancozeb	750
	Xiyang	78% Bordeaux mixture·Mancozeb	500
		20% Pydiflumetofen	2000
August 2021	Chengjiang	70% Sulfur·Thiram·Thiophanate-methyl	750
		68.75% Famoxadone·Mancozeb	750
September 2021	Chengjiang	50% Sulfur·Carbendazim	750
		43% Mancozeb	750
	Xiyang	30% Pyraclostrobin	2500
October 2021	Chengjiang	68.75% Famoxadone·Mancozeb	750
		43% Mancozeb	750
	Xiyang	45% Pyraclostrobin·Thiophanate-methyl	1500
		40% Difenoconazole	2000

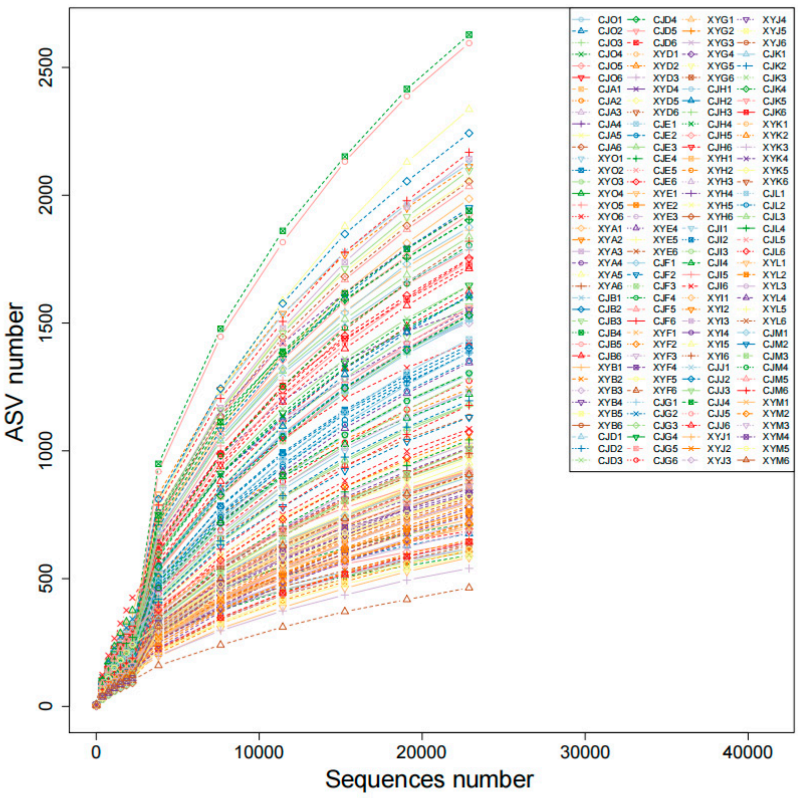

Figure S1. Rarefaction curve of fungi (22,876) in each sample of this study.
